# Snake Robot with Motion Based on Shape Memory Alloy Spring-Shaped Actuators

**DOI:** 10.3390/biomimetics9030180

**Published:** 2024-03-16

**Authors:** Ricardo Cortez, Marco Antonio Sandoval-Chileño, Norma Lozada-Castillo, Alberto Luviano-Juárez

**Affiliations:** 1 Unidad Profesional Interdisciplinaria en Ingeniería y Tecnologías Avanzadas, Instituto Politécnico Nacional, Mexico City 07340, Mexico; nlozadac@ipn.mx (N.L.-C.); aluvianoj@ipn.mx (A.L.-J.); 2 Unidad Profesional Interdisciplinaria de Energía y Movilidad, Instituto Politécnico Nacional, Mexico City 07738, Mexico; masandovalc@ipn.mx

**Keywords:** snake robot, shape memory alloy, hyper-redundant robotics

## Abstract

This study presents the design and evaluation of a prototype snake-like robot that possesses an actuation system based on shape memory alloys (SMAs). The device is constructed based on a modular structure of links connected by two degrees of freedom links utilizing Cardan joints, where each degree of freedom is actuated by an agonist–antagonist mechanism using the SMA spring-shaped actuators to generate motion, which can be easily replaced once they reach a degradation point. The methodology for programming the spring shape into the SMA material is described in this work, as well as the instrumentation required for the monitoring and control of the actuators. A simplified design is presented to describe the way in which the motion is performed and the technical difficulties faced in manufacturing. Based on this information, the way in which the design is adapted to generate a feasible robotic system is described, and a mathematical model for the robot is developed to implement an independent joint controller. The feasibility of the implementation of the SMA actuators regarding the motion of the links is verified for the case of a joint, and the change in the shape of the snake robot is verified through the implementation of a set of tracking references based on a central pattern generator. The generated tracking results confirm the feasibility of the proposed mechanism in terms of performing snake gaits, as well as highlighting some of the drawbacks that should be considered in further studies.

## 1. Introduction

The development of nonclassical robotic systems has been boosted by the increase in application areas where the use of rigid robotic structures is not feasible [1]. There exist several problems of this kind, such as endoscopic applications, the manipulation of objects in restricted spaces, the implementation of prosthesis devices, the exploration of closed spaces, and so on [2,3]. The implementation of robotic structures designed based on biological organisms is one of the fields of research that has shown a high capacity to solve these kinds of problems [4,5].

Of the biological structures that inspire the development of robots, one of the most-used is the anatomy of snakes, which consists of multiple joints with a very limited movement range [6,7,8,9,10]. However, even if each joint only provides a limited range of movement, the combination of all articulations provides a wide range of configurations to modify the shape of the body, thus allowing snakes to maneuver through many kinds of space. Snake robots are considered a promising configuration for the exploration of difficult-to-reach terrain or constrained places in which wheeled robots or even legged configurations may not be useful [11,12]. Typical applications of snake robots include pipe exploration [13,14,15,16]; search and rescue [17,18,19]; exploration, perception, and proprioception [20,21,22,23]; and medical applications [24,25,26,27,28,29], among others.

The robotics based on this kind of architecture are called hyper-redundant, as they possess more degrees of freedom (DoF) than the movement axis in space [30].

There exist several implementations of this kind of robot in the literature, which can be classified based on the way in which their joints are actuated. The first class corresponds to robots that are actuated by the use of classical direct current (DC) motors. These actuators provide a feasible way to control the position of the joints using a closed-loop scheme, but at the same time, they are required to be placed on the robot. As such, the size of this kind of implementation poses problems related to miniaturization and their application in narrow spaces [17,31,32]. The second class implements the use of DC motors that are not placed on the robot, where the force generated by the actuators is transmitted through the use of bands and pulleys that allow the robots to have low diameters such that they may be applied in narrow spaces [33,34,35]. However, the complexity of the control of this kind of actuation system is a main drawback, as the position of the joints is not directly related to the angular position of the motor shaft [36,37]. The third class of robots implements the use of pneumatic and hydraulic actuators, which allow for nonrigid movements and provide a high capacity to manipulate objects; however, at the same time, this kind of robot requires the use of pumps, thus increasing their cost and difficulty of transportation [38,39,40]. The final class of robots implements non-conventional actuators, which can be implemented on the robot without the necessity of a transmission system, such as the parallel elastic actuator [41], hydraulically amplified self-healing actuator [42], and shape memory alloy (SMA) actuators [43,44]. These actuators can be placed on the robot without a significant increase in size, such that they can still be used in narrow spaces. At the same time, it is necessary to consider the nonlinear dynamics of this type of actuator, which pose several challenges regarding their control; in particular, the hysteresis problems is one of their main drawbacks [45].

The use of SMA actuators is one of the main ways to implement this type of robot, as they modify their shape when their temperature increases. This process is generated by the change between a phase when the material has a plastic behavior (called martensite) to a phase that forces a configuration between the molecules of the material (called austenite). They are composed of a metallic material such that they can be warmed through the application of a current between their terminals [46]. This kind of actuator provides a high power/weight relationship that makes them suitable for implementation in robotic structures. At the same time, their capability to be programmed into several shapes makes them versatile for applications requiring several configurations, such as applications in the fields of medicine and biology [47,48,49,50]. The configuration of the shape that the actuator returns requires a programming approach based on a thermomechanical process [51]. The selection of the shape depends on the application of the actuator; however, this poses limitations, as the programming process must be performed previously. One of the drawbacks of this kind of actuator is the degradation from the shape of the SMA due to the effect of constant loads that are applied over cyclical tasks, thus implying that the proposed structure must provide a physical structure that allows for substitution of the actuators once they surpass their number of duty cycles [52,53]. Of the various shapes that could be implemented using SMA actuators, one of the most used is the spring shape. This configuration allows for linear movement in constrained spaces and provides significant force in comparison to the size and weight of the actuator [54]. There are several drawbacks to this kind of actuator, such as the low-speed response related to the thermoelectrical phenomenon that imposes important challenges on the control of the devices where they are applied [55,56,57,58].

The development of a snake robot based on SMA spring-shaped actuators is described in this work, which is based on sections linked with the use of Cardan joints that are modified to use these actuators. To provide feedback to the control law, estimation of the joint angles is performed using magnets and Hall effect sensors. The implementation of a Cardan joint, on which the SMA spring actuators are directly placed, allows for simplification of the way in which the motion is performed and facilitates substitution of the actuators once they start to degrade. The design takes into consideration the implementation of scales on the outside of the links to improve the friction between them and the environment, thus enhancing the interaction between them.

The remainder of this work is structured as follows: In Section 2, the procedure required to program the SMA actuators into a spring shape is described, and the analysis of the sensors required to monitor their performance is detailed. Section 3 provides a preliminary design of the robotic system in order to validate the way in which it performs motion using an agonist–antagonist scheme. In Section 4, the adaptation from the mechanism to generate the robotic system is described, including mechanical changes and electronic circuits. Section 5 presents the snake motion generation algorithm. Section 6 reports the integration and the results, including the implementation of the sensors, the mechanical structure, a description of the way in which SMA compression produces changes in the joints, and the change in shape from the robot based on the SMA effect (i.e., the accumulation of small angular motions between links produces a noticeable shape change, as in biological snakes). In Section 7, a set of conclusions are presented based on the developed work.

## 2. Programming of the SMA Actuators

To obtain spring-shaped actuators from the SMA, it was necessary to apply the process of programming, which consists of the use of a thermomechanical process to fix a predefined shape of the SMA actuator. Through the application of a current input, the programmed shape can be recovered. Selection of the SMA spring shape was performed to implement an agonist–antagonist configuration and, through the shape memory effect, each of the joints of the robotic system was actuated so that the snake robot could interact with the workspace.

The selected SMA to form the spring actuators consisted of a nitinol wire with a diameter of 375 µm. Some important features of the material [59] are given in Table 1. The relationship between the resistance and length of the wire is fundamental for estimating the resistivity of the SMA springs in order to determine the power electronics required for activation of the actuators.

### 2.1. Nitinol Preprogramming

**Figure 1:**
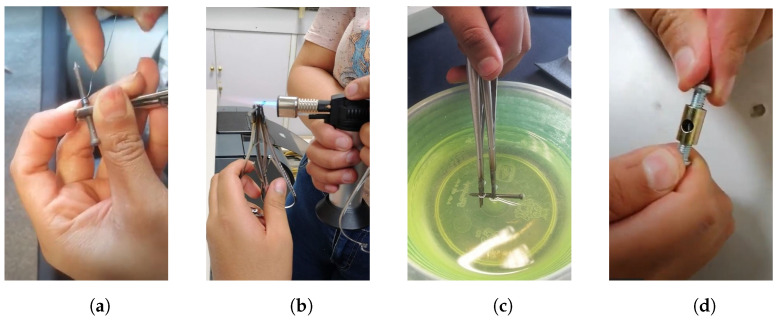
Nitinol preprogramming process. (**a**) Rolling of the SMA spring. (**b**) Preheating of the SMA spring. (**c**) Cooling with water. (**d**) Mechanical constraint.

This process consisted of carrying out manual manipulation of the nitinol wire, followed by a heat treatment to obtain the desired shape, as detailed in the following steps:

1. Using a Matthews clamp, one end of approximately 1.5 cm of the proximal portion of a 20 cm long nitinol wire was attached to a 2.8 mm diameter cylindrical metal structure, thus verifying that it was firmly immobilized. The rest of the cable was wound up manually while taking care that the generated turns were aligned and without any space between them. Using another Matthews clamp, the distal end was grasped, and, thus, the shape of the spring was secured in the metal barrel (see Figure 1a).
2. Heat was applied to the spring, which was generated in the metal cylinder using a torch. During the application of heat, it was visually validated at all times that the material did not denature, which occurs if the wire presents a color change to white. The application of heat was stopped once a homogeneous color change to a copper color throughout the entire length of the spring was observed, as depicted in Figure 1b.
3. A tub of water was used to dissipate the heat from the clips and spring such that it could be removed from the cylindrical metal frame (see Figure 1c). The remaining water was then removed from the spring in order to avoid rust forming on the material during the following stages.
4. Finally, the spring was placed inside a structure as a mechanical constraint in order to ensure the conservation of its shape in the annealing process (as shown in Figure 1d).

### 2.2. Annealing

**Figure 2:**
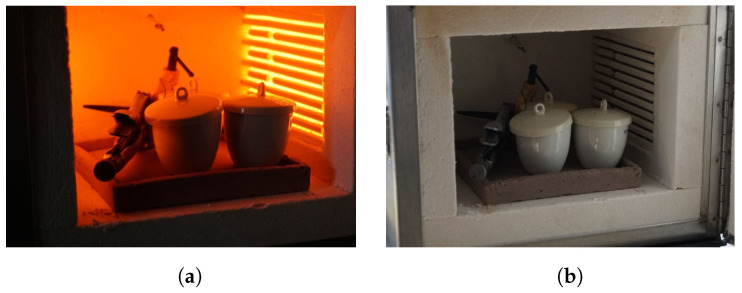
SMA annealing procedure. (**a**) Heating process using the muffle. (**b**) Passive dissipation of the crucibles.

The springs were placed in crucibles that were introduced into an electric muffle for 5 h at 500 °C (see Figure 2a). To reach this temperature, a positive slope from the ambient temperature to the annealing temperature was performed over a hour. Then, the muffle was turned off and the gate was opened, thereby allowing for passive heat dissipation (as shown in Figure 2b). This process required at least 4 h in order to ensure that the crucibles reached ambient temperature before their removal from the muffle.

**Figure 3:**
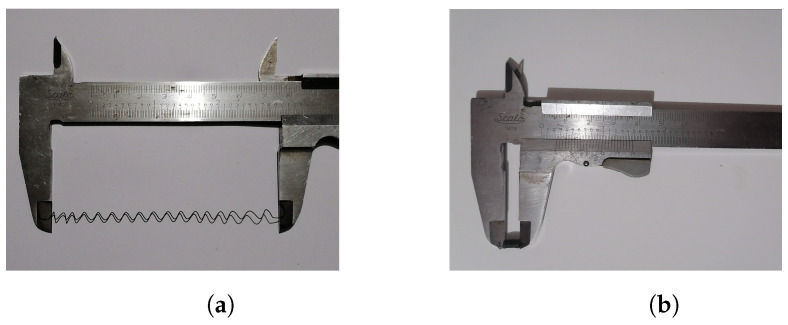
Resulting shape of programmed SMA springs. (**a**) Expanded SMA spring. (**b**) Programmed shape corresponding to a compressed spring of the SMA.

In consequence, 7 mm long compressed SMA springs were obtained, as depicted in Figure 3a, with an elongation capacity of up to 9 cm without losing their capability to restore their shape when heated (see Figure 3b). The length of the compressed SMA springs allowed for the design of links where the joint space possessed a minimum size of 7 mm for placement of the actuator but, at the same time, the movement of the agonist–antagonist mechanism requires at least double the amount of space to be noticeable. As the maximum elongation exceeded the 14 mm required, the SMA actuator was considered feasible for implementation in the robotic system.

### 2.3. Sensors Associated with the SMA Actuators

The main variables associated with each SMA actuator for the development of a controller are the displacement of each SMA spring and the internal temperature of the SMA wires. The relationship between the temperature and the force generated by the actuator has been given in [54] and, as such, this variable can be related to the capability to modify the displacement over the joint.

As the direct displacement of the SMA spring is not a variable of interest in the robotic system, the position measurement was performed over the joint to obtain an angular position that indirectly depends on the length of the spring. This position measurement was performed using an SS49E Hall effect sensor due to its characteristics, such as its capacity to sense the magnetic field and its polarity, its small size (5 mm diameter) that makes it suitable for implementation on the robot, and its low power consumption of 30 mW. This sensor was implemented in combination with neodymium magnets (1500 Gauss), which were placed on the joint that moves by spring compression.

The voltage response of the Hall effect sensor, as a function of the magnetic field that is dependent on the distance to where the magnet is located, was characterized through polynomial interpolation. As the sensor responds to positive and negative Gauss magnitudes, the poles of the magnets must first be identified. Then, the north magnetic pole of the magnet was aligned with the front face of the sensor, as this arrangement allows for distinguishing between the movements in each of the two degrees of freedom. The detailed procedure of the process can be found in [60]. In this particular case, the method was simplified for the use of only two sensors.

**Figure 4:**
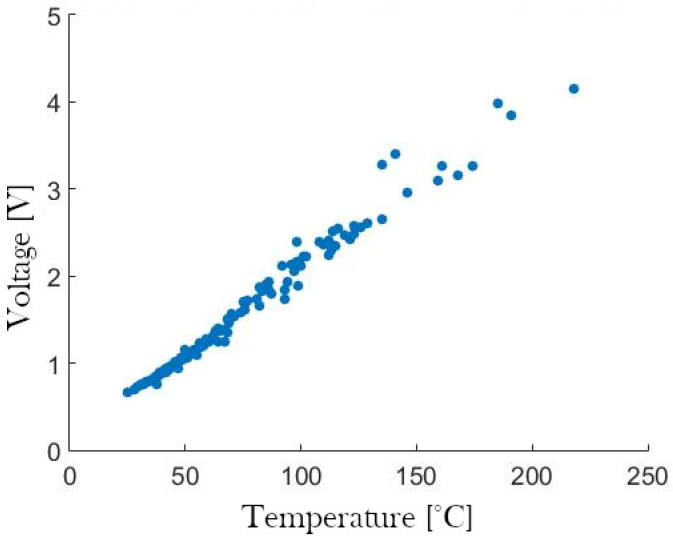
Characterization of the shunted thermistor.

The temperature of the SMA springs was measured using NRBE104F3435B2F Eaton thermistors, which have an operating interval that was considered adequate for the application (this choice was made following the technical recommendations provided in [61]), an affordable acquisition cost, and a suitable size for implementation inside the robotic system. Due to its nonlinear response, a linearization around the operating interval had to be performed, which was based on a shunted thermistor approach [62] for the temperature interval from 23 to 125 °C. The resulting characterization of the linearized thermistor is depicted in Figure 4.

The acquisition of each of the 12 thermistors required to sense the temperatures of the SMA springs in the robot involved the design of a circuit integrating the CD74HC4067 16-channel analog multiplexer/demultiplexer in order to optimize the available pins of the microcontroller without information loss, as the maximum delay time of the switching process corresponds to 0.5 ms, which is lower than the sampling rate of 10 ms used for data acquisition. The circuit was designed using the Autodesk Eagle (Fusion 360) software and consisted of two CD74HC4067 circuits, and the same method was applied to process the analog data measured by the SS49E Honeywell Hall effect sensors.

## 3. Computer-Aided Preliminary Design

**Figure 5:**
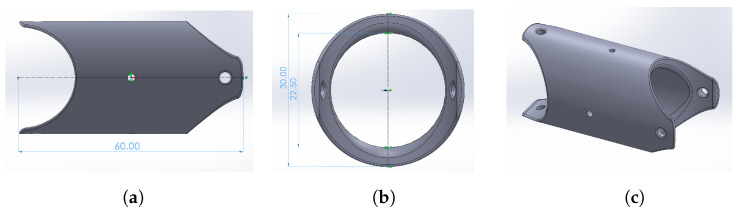
Modular link design. (**a**) Lateral view. (**b**) Frontal view. (**c**) Isometric view.

Using the dimensions of the SMA springs as a basis, modular links joined by rotational joints that allow for mobility in two degrees of freedom were proposed. The structure was inspired by the universal joint design for the redundant robots of Evangeliou and Tzes [63]. This configuration allows for complementary and antiparallel coupling between links from two rotational axes using a simple design articulation. A modular link structure with 2 DoF having mutually perpendicular axes was designed with an external diameter of 30 mm for a cylindrical link, an internal diameter of 22.5 mm, and a length of 60 mm, whose extrusions were adjusted such that a mobility of at least 20 degrees was ensured in each degree of freedom. The CAD of the structure is shown in Figure 5.

A rotational joint was designed to allow movement in 2 DoF based on the Cardan design. The piece consisted of a toroidal disc that is 7 mm thick and 22 mm in diameter. It has a central hole for 10 mm diameter wiring. The last piece fulfills two functions:

- It articulates the links with each other in order to allow the motion of the joint.
- It acts as an anchoring surface for the springs when installed in the median plane transversely in the links.

**Figure 6:**
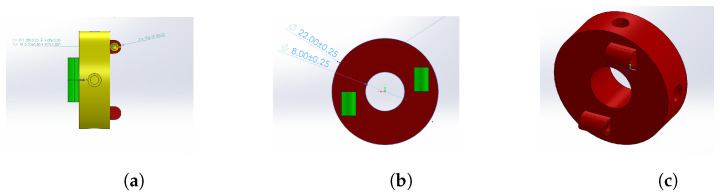
Rotational joint design. (**a**) Frontal view. (**b**) Lateral view. (**c**) Isometric view.

For the mechanical anchoring of the SMA springs, two pairs of semicylindrical extrusions with countersunk holes were added, which were arranged on opposite axes and perpendicular to each other (see Figure 6).

The mechanical articulation of the joint and the links involved the design of an interconnection hole, which was inserted in the slot of the cylindrical face of the joint, in the anchorage slots of the link, and fixed with a countersunk screw towards the joint.

**Figure 7:**
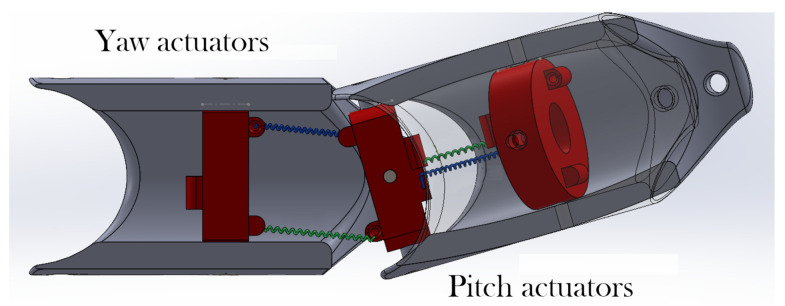
Assembly with SMA actuation.

Figure 7 shows the assembly for two links articulated by a rotational joint. This represents the configuration of the SMA actuators anchored to their respective joints and allows for mobility in two degrees of freedom of the subsequent link, where the articulation is actuated by the complementary pairs of SMA springs. The actuators on the left control the movement in the perpendicular axis (yaw), while actuators on the right side control the movement in the horizontal axis (pitch). Both configurations work in an agonist–antagonist fashion.

**Figure 8:**
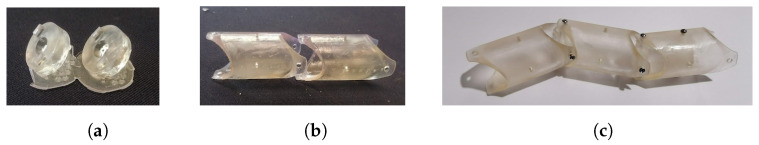
Resin 3D-printed mechanism. (**a**) Printed joint. (**b**) Printed links. (**c**) Main assembly.

The 3D printer used to manufacture the pieces was a Phrozen Sonic Mighty 4K, with a maximum printing resolution of 52 µm and a thickness of the printing layers from 0.01 to 0.3 mm. The software used for layer segmentation of the pieces was Chitubox Free 1.7.0. The material selected for 3D printing of the modules was a high-temperature resin (Phrozen TR250LV). Due to its capacity to resist high temperatures, it was considered an appropriate material to avoid any melting effects, despite being in direct contact with the SMA actuators. A first implementation of the design is shown in Figure 8, which allowed us to evaluate whether the proposed design allowed for movement between the links once assembled. At the same time, it was noticed that the disk was not suitable for assembly using this procedure, as it lacked the required details for implementation of the joint.

### 3.1. Mechanism Adaptations

After a performance assessment of the printed mechanism, the following modifications were proposed to improve the design:

1. To facilitate assembly of the robot, the dimensions of the links were increased by 25%. The robot assembly approach was also modified by segmenting the proximal, modular, and distal links according to the median plane of its longitudinal axis, which was carried out in addition to reducing the weight of the mechanism derived from the increase in dimensions.
2. For the new assembly approach, a distinction was considered between the rotational joints that interconnected the links from internal spring anchor joints.

### 3.2. Modular Link

**Figure 9:**
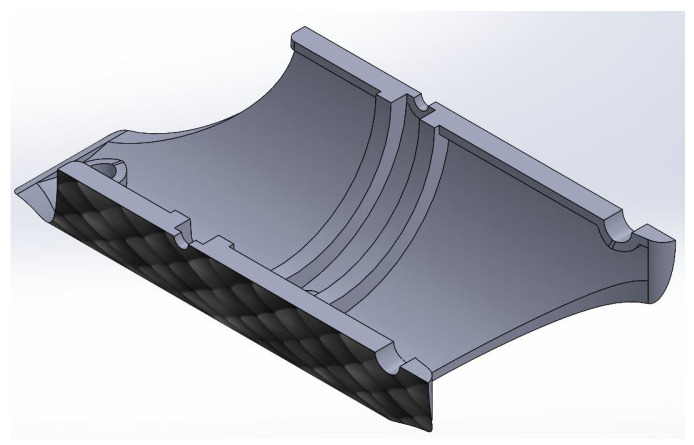
Link with longitudinal cut in the middle plane.

Regarding the link presented in Figure 5, the thickness of the link was decreased from 3.7 to 2 mm, and an arch was extruded in the median plane of the transversal axis to seat the disc that interconnects the links (see Figure 9). To increase the coefficient of friction of the outer surface of the link, a scaled texture was added.

### 3.3. Proximal Link

**Figure 10:**
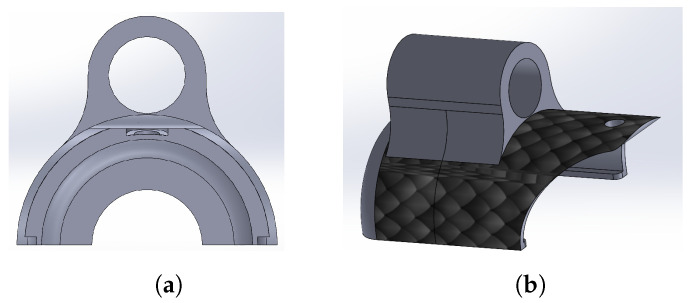
Proximal link design. (**a**) Front view. (**b**) Isometric view.

The proximal link was designed to mate with the linear actuator and thus transmit the linear motion to the robotic system for its insertion into the environment to be explored. From the link presented in Figure 9, a cylindrical extrusion was made on the outside with a 0.9525 mm diameter hole for insertion of one of the axes of the linear actuator (see Figure 10).

**Figure 11:**
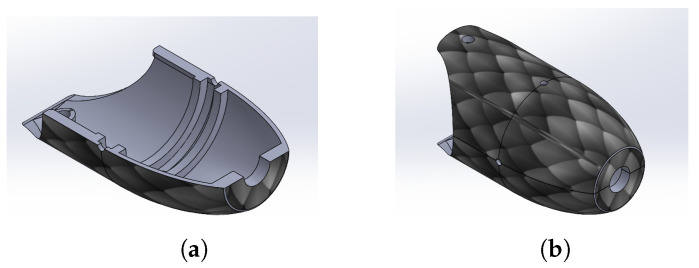
Distal link design. (**a**) Front view. (**b**) Isometric view.

From the median transverse plane of Figure 9, a rounded cover was generated to form the distal link, as depicted in Figure 11.

**Figure 12:**
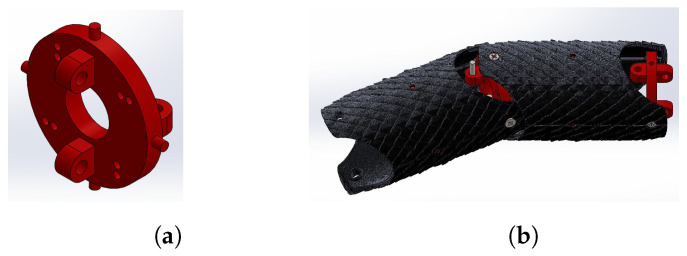
Rotational joint assembly. (**a**) Joint with anchorage. (**b**) Two links assembly.

The rotational joint was used to articulate links with each other using four holes in the outer face of the toroidal disc. The thickness of the proposed joint was 4 mm with a diameter of 27.5 mm. For mechanical anchoring of the SMA springs, two pairs of semicylindrical extrusions were added. Likewise, a disc was designed to establish anchor points in the transverse median plane of the links, thus generating four additional extrusions on the outer face of the toroidal disk, as depicted in Figure 12a. Figure 12b shows the assembly with the rotational joint.

### 3.4. The 3D Printing of the CAD Proposals

**Figure 13:**
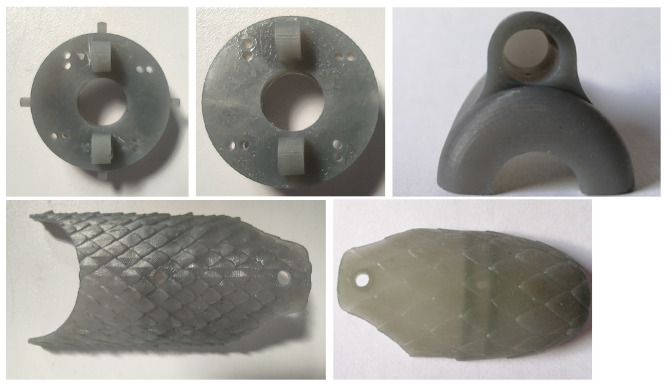
Resin CAD-printed pieces.

Using the printer and resin stated at the beginning of this section, the CAD designs shown in Figure 13 were printed, thereby obtaining the parts for assembly of the final prototype of the robot.

Switched activation strategies can be used for nitinol, as the activation of the spring (actuator with SMA) can be on or off [64]. The programmed actuators require a current of 1 A for activation. A pulse width-modulated (PWM) signal was used to control the current supplied to the actuator.

**Figure 14:**
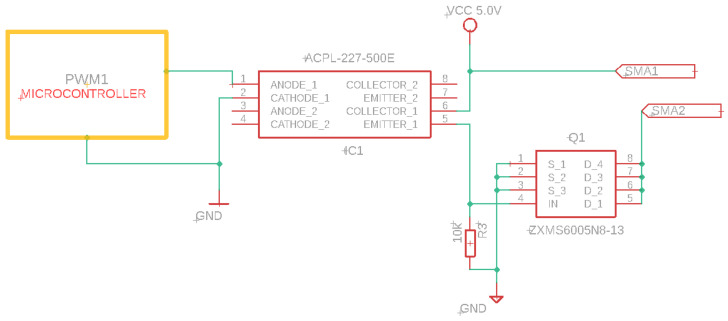
Schematic of the activation circuit of the SMA actuator.

For the power supply, a power electronics circuit similar to the one proposed in [43] was used. A transistor-based optocoupler isolates the power stage from the control device, and an output signal switches to a fast-operating (5 µs delay) N-channel MOSFET transistor (see Figure 14).

The activation of each of the 24 SMA springs required in the robot required a circuit to integrate and multiplex each stage for a single actuator.

## 4. Snake Robot Model

### 4.1. Kinematic Model

The model for the snake robot was proposed based on the Denavit–Hartenberg convention that allows for simplification of the description of a robot and its kinematics through the definition of a set of parameters (given in Table 2) that allows for the determination of the existence from a rotational joint composed of two rotational joints with reference frames that are normal between themselves. It should be noted that the odd joints correspond to horizontal movements, and even joints correspond to vertical movements of the robot.

**Figure 15:**
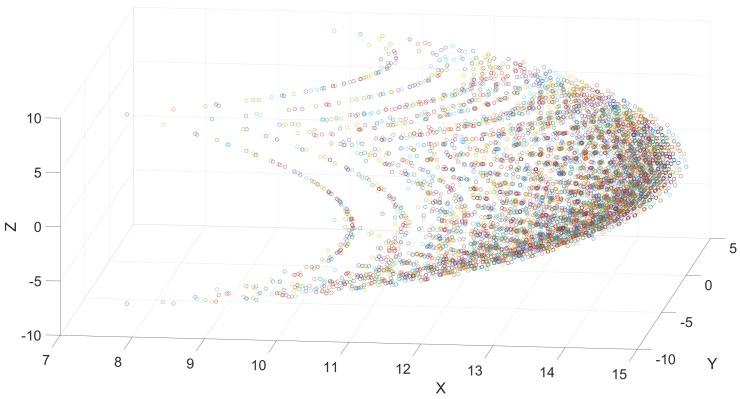
Snake robot distal position workspace based on Denavit–Hartenberg convention.

This provides a set of feasible positions from the distal point of the robot in the workspace that is reachable by an angular motion in the range $\left[-20^{\circ },20^{\circ }\right]$ for each joint from the robot, as shown in Figure 15. It can be noted that, once the limited angles for each joint are modified, the resultant workspace provides a wide area that can be reached.

### 4.2. Dynamical Model

**Figure 16:**
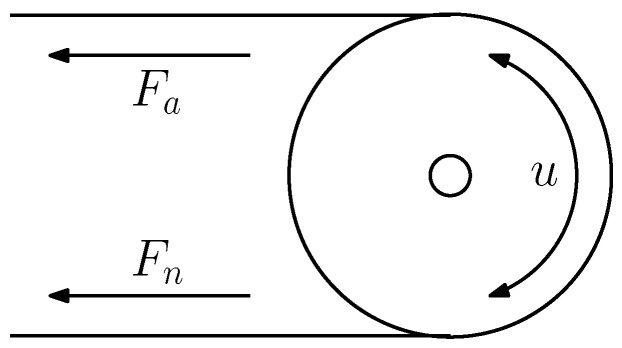
Agonist–antagonist scheme for motion of the robot’s joints.

To avoid the problems related to the modeling of robots that possess a high number of DoF, we produced complex dynamical models for each joint—considered as an independent system to be controlled—where the forces generated by the connections between links are considered as a perturbation to be compensated for by the controller. The dynamical model for the robot’s independent joints is defined as follows:

(1) $$\begin{array}{ccc} \ddot{\theta } & = & f(\theta ,t,d)+g(u,t)\\ y & = & h(\theta ,\dot{\theta },t) \end{array},$$

where θ corresponds to the angular position from each joint, the external disturbance that affects the robot (e.g., gravity and friction forces) is defined as *d*, and *u* is the force generated by the SMA actuators, which is defined as

(2) $$u=F_{a}-F_{n},$$

where $F_{a}$ and $F_{n}$ correspond to the agonist and antagonist forces generated by the SMA springs, respectively. The way in which this is related to an angular motion is depicted in Figure 16, which shows how the linear forces generated by contraction of the spring are applied as an angular force for displacement of the joint.

The way that this force is generated depends on the SMA derivative temperature, which is defined as follows [54]:

(3) $$F=h\left(T,\frac{dT}{dt}\right)=\left\{\begin{array}{cc} \frac{a_{l}}{1+e^{-b_{l}\left(T-d_{l}\right)}}+c_{l} & \frac{dT}{dt}<0\\ \frac{a_{u}}{1+e^{-b_{l}\left(T-d_{u}\right)}}+c_{u} & \frac{dT}{dt}\geq 0 \end{array}\right.,$$

where the terms $a_{l},b_{l},c_{l},d_{l},a_{u},b_{u},c_{u},d_{u}\in R^{+}$ are parameters that define the relationship between the generated force and the input temperature *T*. The terms $a_{l}$ and $a_{u}$ represent the maximum force generated by the springs, $b_{l}$ and $b_{u}$ are related to the change rate of the forces when the temperature is modified, the terms $c_{l}$ and $c_{u}$ correspond to a residual force generated by the springs due to their stiffness, and finally, the terms $d_{l}$ and $d_{u}$ are related to the activation temperature of the springs.

The dynamic of the temperature is given by

(4) $$\frac{dT}{dt}=\alpha I^{2}-\beta \left(T-T_{a}\left(t\right)\right),$$

where α is a term related to the heat gain from the material, the term β corresponds to a dissipation heat parameter, and $T_{a}$ is the ambient temperature, which modifies the operational temperature of the springs. The maximum and minimum temperatures of the spring are given by

(5) $$\begin{array}{ccc} T_{\min} & = & T_{a}\\ T_{\max} & = & \frac{\alpha I^{2}}{\beta }+T_{a}. \end{array}$$

It should be noticed that, as the force generated by the springs depends on the change of temperature, a drawback of the implementation is that it may produce errors when a high-speed reference is proposed. This is because even if the heating could be performed by a current increase, the cooling process depends on the natural dissipation of heat from the material, thereby limiting the feasible tasks that may be performed without the addition of a cooling system.

## 5. Snake Motion Implementation

### 5.1. Central Pattern Generator for Snake Gait

**Figure 17:**
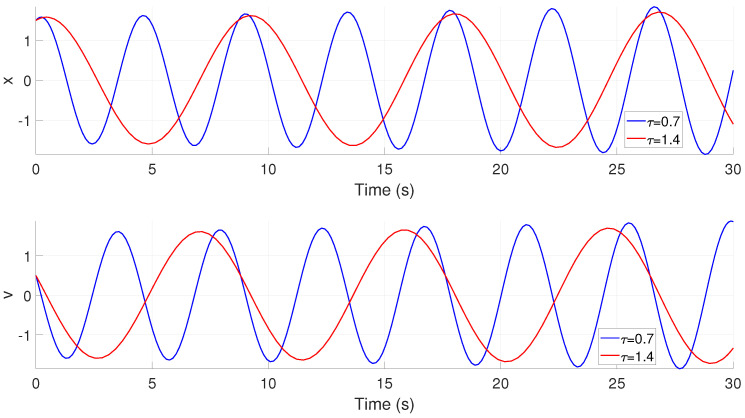
Dynamical oscillator based on (6), which modifies the generation of a sinusoidal gait for rectilinear crawling and lateral undulation.

The robot is required to follow a motion gait based on the two classical movements of snakes—lateral undulation and rectilinear crawling—based on a set of sinusoidal angle joints that are propagated over the body of the robot in the horizontal or vertical joints, respectively [65]. A central pattern generator (CPG) was proposed to compute the trajectories to be performed over the joints of the robot, which is based on a set of oscillators with the following structure [66]:

(6) $$\begin{array}{ccc} \dot{v} & = & -\alpha \frac{x^{2}+v^{2}-u}{\tau u}v-\frac{x}{\tau }\\ \dot{x} & = & \frac{v}{\tau } \end{array},$$

where *v* corresponds to the velocity of the joint, and the angular position from the joint is given by *x*. The parameter α modifies the way in which the oscillations are performed from the convergence velocity to the stable oscillation, the term τ changes the frequency of the oscillator, and the parameter *u* defines the amplitude of the oscillations, which is given by $\sqrt{u}$. The resulting sinusoidal trajectory is shown in Figure 17, from which it can be seen that the frequency of the performed trajectory is modified based on changes in the τ term; namely, when this parameter is increased, the velocity of the oscillator decreases.

**Figure 18:**
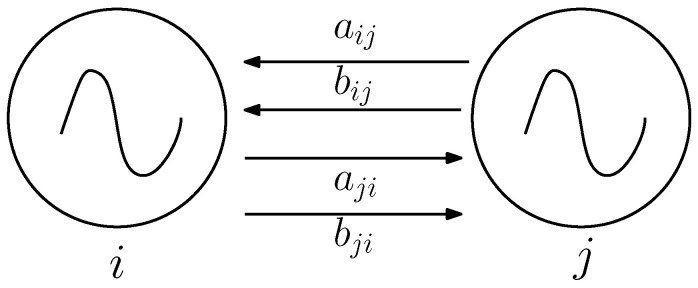
Connection between oscillators to perform synchronization.

This kind of oscillator produces a unique oscillation that corresponds to a single joint angle. In order to implement a CPG, it is necessary to define an oscillator that could be linked with another oscillator to ensure synchronization between the oscillators, thereby ensuring that the desired gait is performed when tracking.

(7) $$\begin{array}{ccc} {\dot{v}}_{i} & = & \dot{v}=-\alpha \frac{x^{2}+v^{2}-u}{\tau u}v-\frac{x}{\tau }+\sum_{j}\frac{a_{ij}x_{j}+b_{ij}v_{j}}{\tau }\\ {\dot{x}}_{i} & = & \frac{v}{\tau } \end{array},$$

were the terms $a_{ij}$ and $b_{ij}$ correspond to the weights of the connections between oscillator *i* and oscillator *j*, respectively, as shown in Figure 18, from which it can be seen that there exists a two-way connection between them.

**Figure 19:**
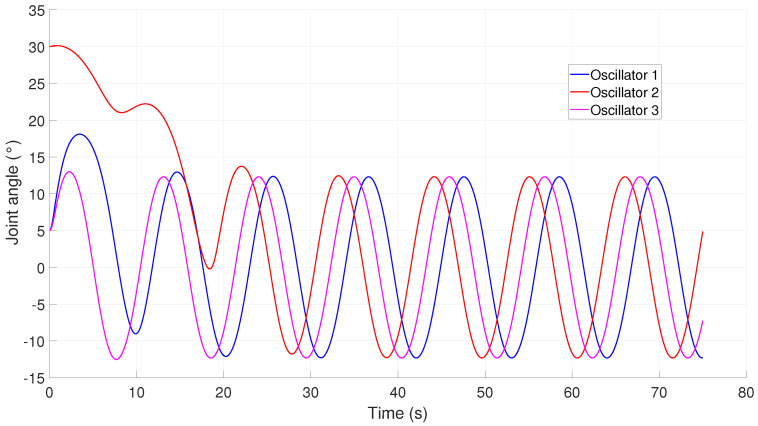
Joint angle trajectories generated by a CPG.

To generate the reference signals for the robot, a total of three oscillators were simultaneously implemented, with connections between all of them to ensure their synchronization. This enabled a set of tracking references to be obtained, as shown in Figure 19, which could be implemented for lateral undulation or rectilinear crawling based on whether they were applied to the horizontal or vertical joints of the robot. It can be noted that during the first few seconds of the dynamical system, the CPG produced trajectories that were not suitable for motion; however, once the oscillators had synchronized, they produced a set of references that could be implemented for the snake gait motion.

### 5.2. Agonist–Antagonist Control Scheme

To implement control of the joints on the robot, a closed-loop scheme in two stages had to be proposed. The first stage corresponds to the control of the joint based on selection of the SMA spring to be activated, which is defined as follows:

(8) $$u_{a}=\left\{\begin{array}{cc} Ke & \mathrm{if}\;e>0\\ 0 & \mathrm{if}\;e\leq 0 \end{array}\right.,$$

(9) $$u_{n}=\left\{\begin{array}{cc} 0 & \mathrm{if}\;e>0\\ K|e| & \mathrm{if}\;e\leq 0 \end{array}\right.,$$

where e=x−θ corresponds to the tracking error for the joint motion, thereby allowing for selection of the SMA spring that must be activated to reach the desired position, and *K* corresponds to a proportional gain.

The second stage considers that the force from the springs is generated by a change in temperature, as defined in (3). To generate this change in temperature, the signal controls $u_{a}$ and $u_{n}$ must be converted to a current that modifies the thermal dynamic, as given in (4) by the following structure:

(10) $$u_{s}=\frac{100}{1+e^{\left(100-u\right)},}$$

where *u* corresponds to the computed control signal—$u_{n}$ or $u_{a}$. The computed $u_{s}$ is limited to the range (0,100) and corresponds to a pulse width modulation (PWM) value that could be applied by a digital microcontroller and a power electronic stage.

## 6. Integration and Results

### 6.1. Mechanical and Instrumentation Implementation

**Figure 20:**
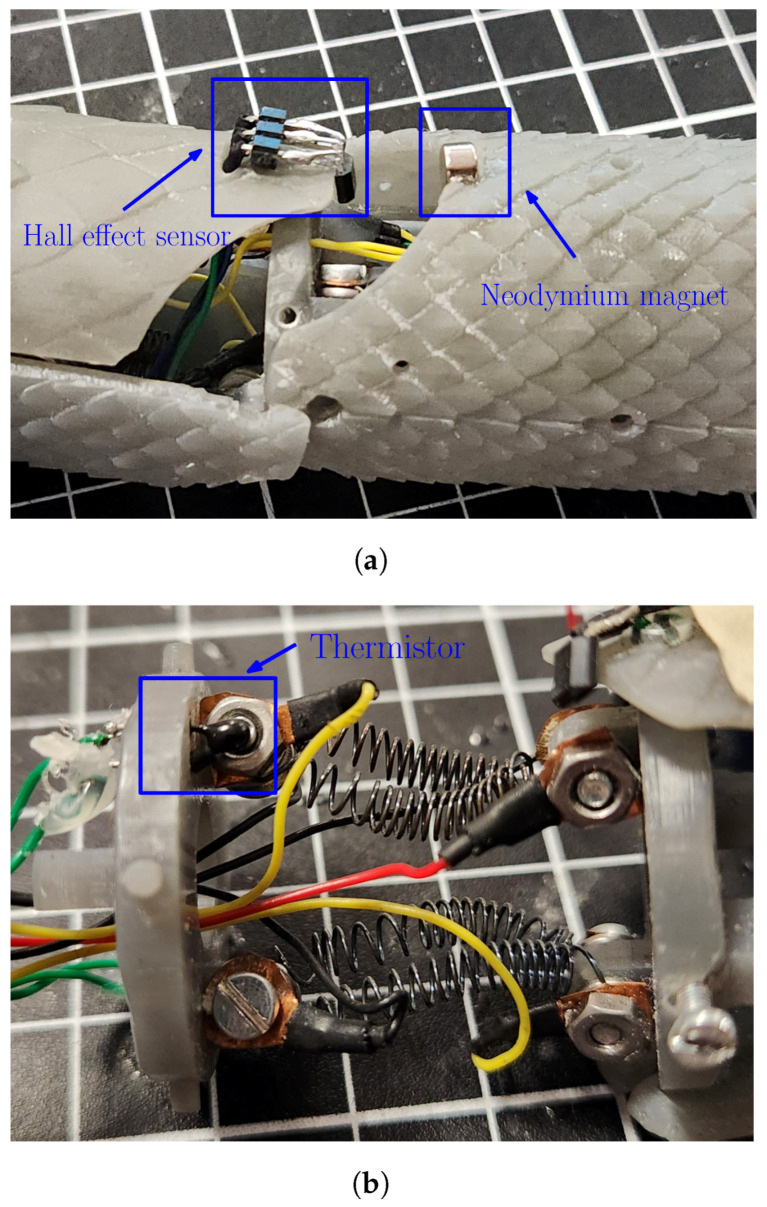
Instrumentation for state monitoring of SMA joint mechanism. (**a**) Hall effect sensor in configuration with the neodymium magnet for position estimation. (**b**) Thermistor used as temperature sensor for the SMA spring.

The implementation of the sensors in the robotic system is presented in Figure 20, from which it can be noticed that the placement from the Hall effect sensor is close to the neodymium magnet in such a way that the measured value from the magnetic field could be used to estimate the angular position of the joint over the corresponding axis. At the same time, the placement of the thermistor can be seen in Figure 20b; in this case, the sensor must be in contact with the metallic surface to estimate the temperature of the spring.

**Figure 21:**
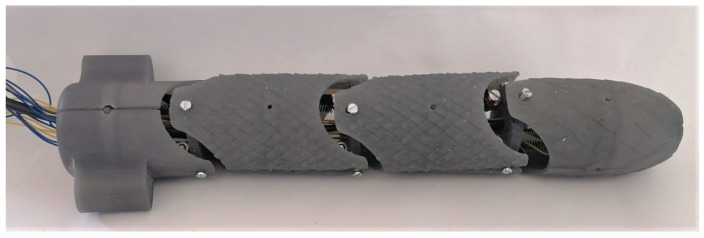
Four-link robot assembly.

Figure 21 shows a four-link snake robot assembly without the borescope camera and the prismatic joint, which allows us to notice that the mechanical design allows for the configuration of links and joints into a close structure. At the same time, avoiding the use of classical actuators (e.g., DC motors) allowed for a reduction in size and autonomous functionality, as the robot does not require a connection to a platform that contains the actuators in order to transmit the movement by bands or wires; the only requirement is connection to the electronic stage, thereby simplifying their application in several tasks. It should be noted that regarding this point, the robot can work independently of the linear actuator mechanism; however, this was implemented to provide its controlled introduction into narrow spaces.

**Figure 22:**
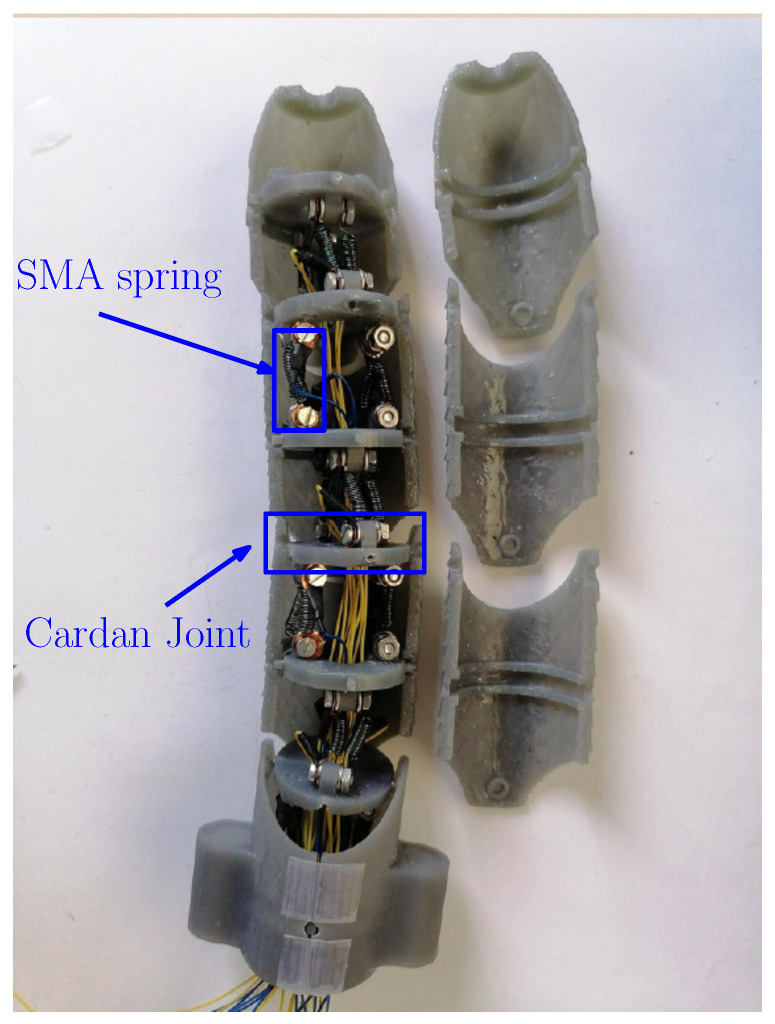
Inside view of the robot, showing the Cardan joints and the placement of the SMA actuators.

The internal mechanical structure of the robot is shown in Figure 22, from which the placement of the four SMA springs and their connections with the Cardan joints that allow for 2 DoF movement between links can be seen. The agonist–antagonist mechanism is visible, as the way in which they are placed implies that if the agonist compresses, then the antagonist suffers an elongation (and the same happens if the antagonist is the one that compresses by returning to its programmed shape).

**Figure 23:**
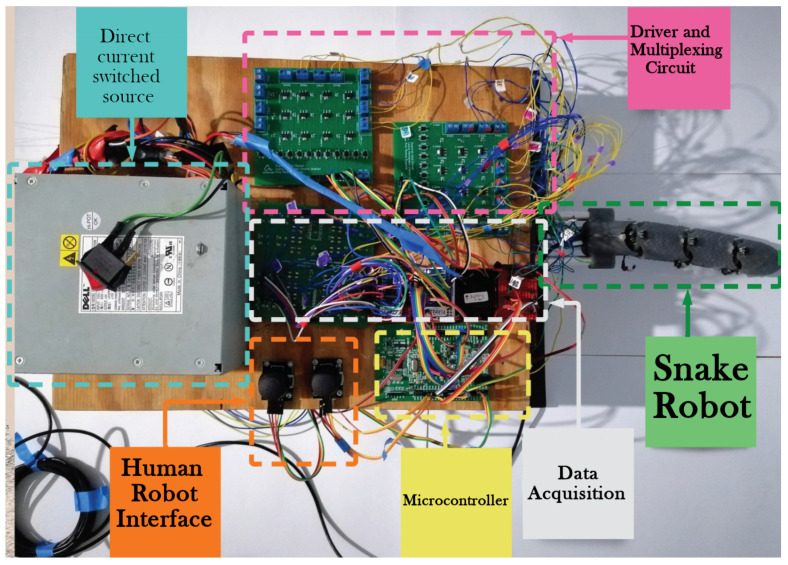
Integrated system.

The integration of the complete robotic system and the way in which the connections are performed is detailed in Figure 23, regarding which the following notes are presented:

- Direct current switched mode: This consists of a 12 V voltage source with a power of 120 Watts to provide sufficient current for the SMA actuators.
- Driver and multiplexing Circuit: This provides the implementation of the analog multiplexers to enable measurement of all the required signals by the sensors with a minimum number of analog channels.
- Snake robot: This generates movements through the use of SMA spring actuators while at the same time retrieving information of their state using temperature sensors and Hall effect sensors to determine the angular position of the joints.
- Power stage: This allows for electronic activation of the SMA actuators via the implementation of PWM signals to regulate the current, which is provided through the use of a MOSFET switching configuration. This stage is optoelectronically isolated in order to avoid any damage produced by malfunctioning.
- Human–robot interface: This consists of two joysticks, one of which provides the user the capability to modify the positions of each joint of the snake robot, while the other allows for the control of the linear actuator mechanism.
- Microcontroller: Facilitates the data acquisition of the sensors and a communication channel with the PC, thereby allowing a graphical interface to generate corresponding computations. At the same time, it receives information from the PC to generate the PWM signals required to control the SMA actuators.

**Figure 24:**
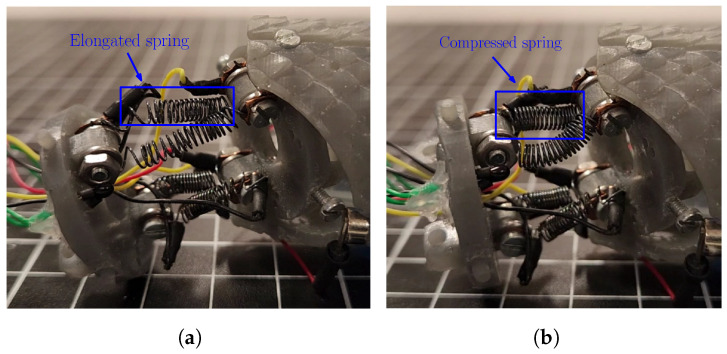
Comparison between the joint assembly with the use of SMA springs for elongated and compressed states of the spring. (**a**) Elongated SMA spring. (**b**) Compressed SMA spring.

To test the motion capability of the robotic system, a set of tests was performed to determine its ability to perform an exploration task. The first test consisted of testing of a single Cardan Joint detached from the robot in order to exemplify the way in which the SMA springs generate the motion of the joint (see Figure 24). Compression of the spring due to the memory effect produced by the change between the martensite and austenite phases of the material leads to motion of the joint, thus modifying the relationship between the links.

### 6.2. Angular Motion Test

**Figure 25:**
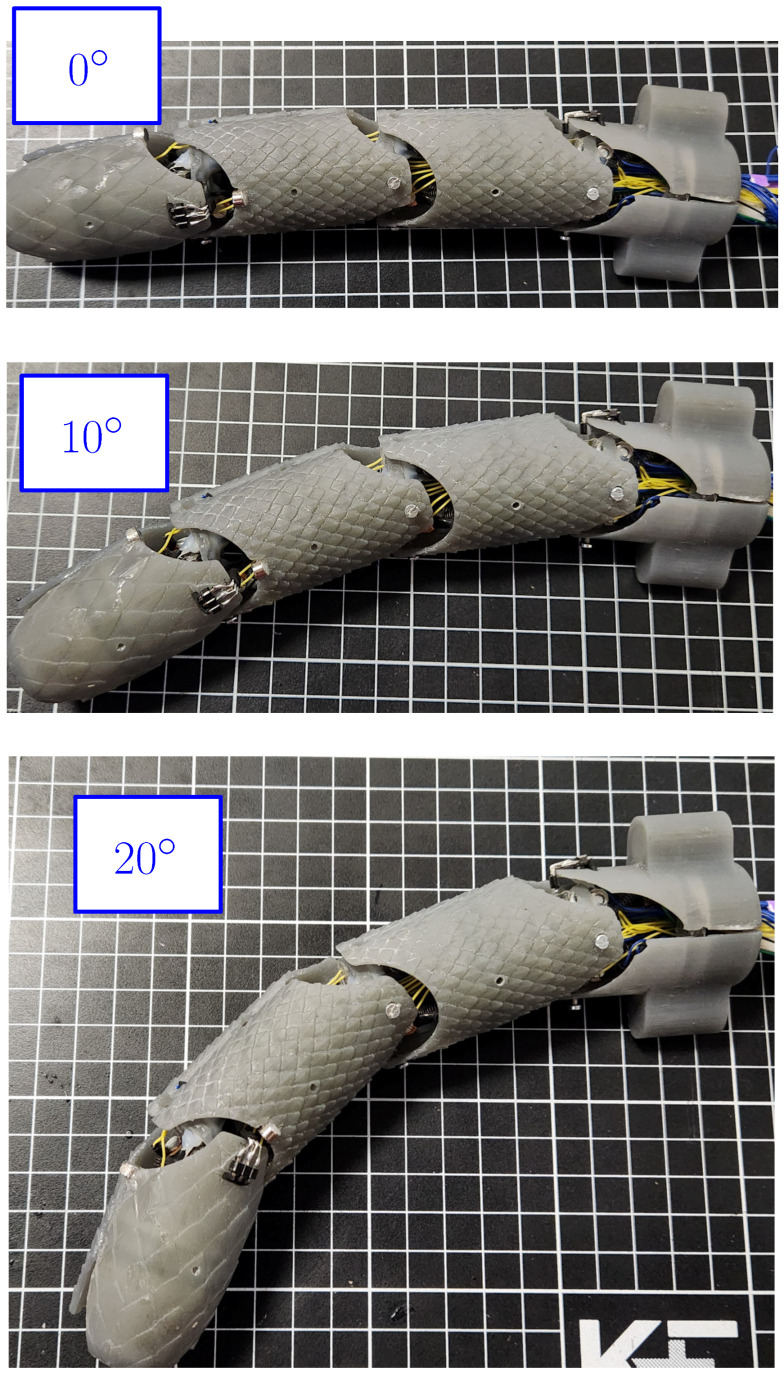
Movement test of the horizontal joints with different angles between links.

Next, a test was performed to verify the motion of the links and the way in which they modify the robot shape. The motion was assessed by considering different angles obtained by the SMA actuators. The test is depicted in Figure 25, where all joints share the same angle to exemplify the way in which the shape of the robot is modified. In the first case, the “a” angle corresponds to $0^{\circ }$, and it can be noticed that while the robot is almost straight, a slight deviation was produced by the way that the SMA actuators were placed inside the robot. In the second case, a value of $10^{\circ }$ was implemented on each joint, and it can be noticed that the robot tended to modify its shape. In the third case, the joints took a value of $20^{\circ }$, thus providing a clear change in the shape of the robot. The reason for the noticeable differences between the cases is the accumulation of small changes between links producing a noticeable change in shape, as in biological snakes. It must be noticed that the velocity from the joints is limited to 7 degrees per second. This is related to the SMA response time for the actuator, which requires approximately two seconds of heating to reach the temperature where the austenite phase generates the shape change. This time could be reduced by the increase of the current applied to the SMA spring to improve the heating, but it requires a modification from the power electronics used for the control of the actuators.

**Figure 26:**
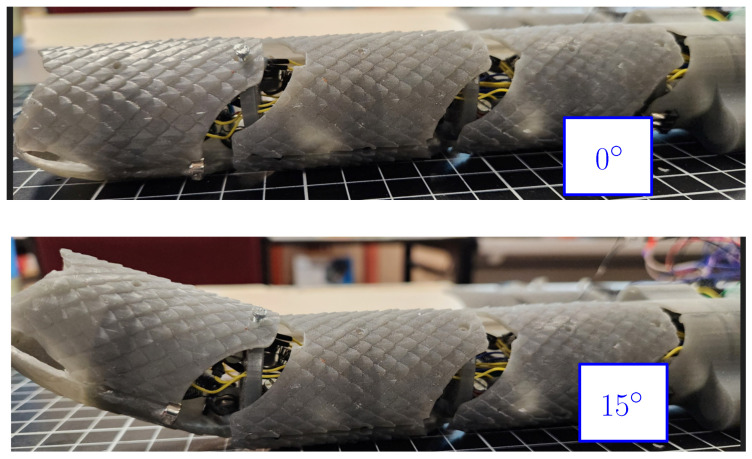
Movement test of the vertical joints with different angles between links.

Finally, a test was performed to verify that vertical motion could be achieved by activation of the SMA springs. Figure 26 shows the motion in the distal link produced by compression of the SMA spring. In this particular case, it is expected that each joint is capable of carrying one link, as the environmental conditions of their application do not require further effort.

### 6.3. Snake Gait Motion Test

To evaluate the way in which the robot is capable of carrying out motion based on a snake gait, two sets of experiments were performed based on the trajectories computed by a CPG (once the oscillators had been synchronized in order to avoid nondesired trajectories). The first set of tests considered the application of trajectories over the horizontal joints in order to perform a lateral undulation gait, while the second set considered the application of reference trajectories over the vertical joints to generate a rectilinear crawling gait. In each case, the controllers of the nonused joints were set to a value of zero in order to avoid the complex movements producing nondesired perturbations.

**Figure 27:**
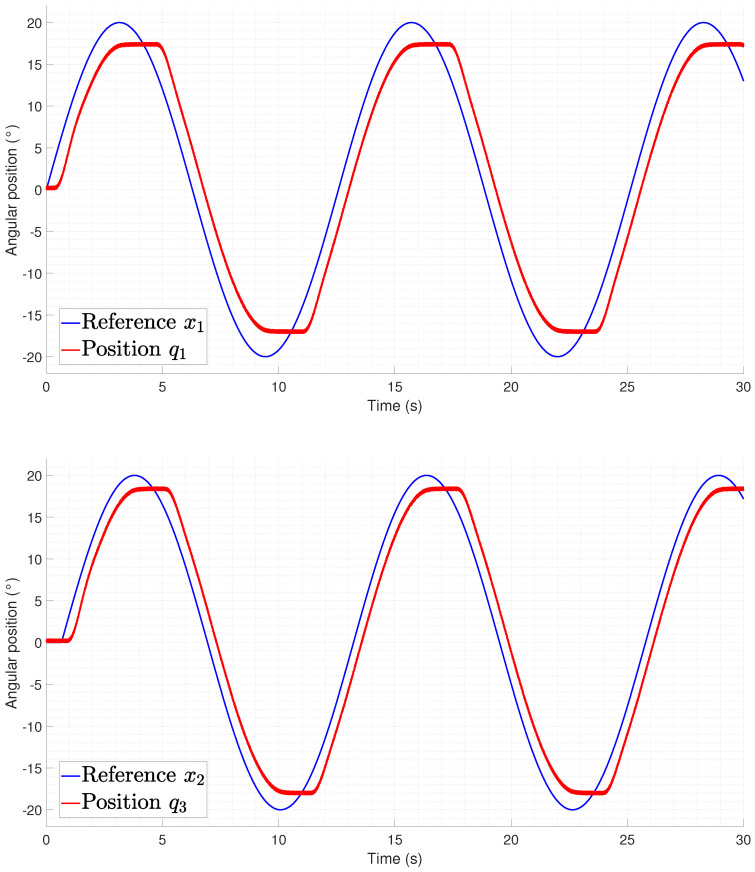
Tracking of odd joints during a lateral undulation gait pattern ($q_{1},q_{3}$).

**Figure 28:**
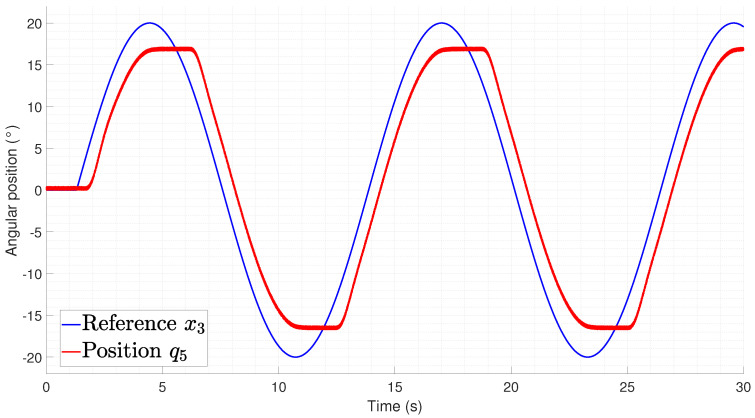
Tracking of odd joints during a lateral undulation gait pattern ($q_{5}$).

Figure 27 and Figure 28 show the way in which the lateral undulation was performed, and it can be seen that the motion from the joint was not performed at the same time as in the provided reference. This phenomenon is related to the necessity of heating and cooling the springs to generate the desired motion, which was mainly noticed when there was a change of direction in the reference. The differences between the required time for initializing the motion for each joint depended on the heating and cooling of the agonist–antagonist system in order to generate a force that could compensate for the mechanical dynamics of the robot.

**Figure 29:**
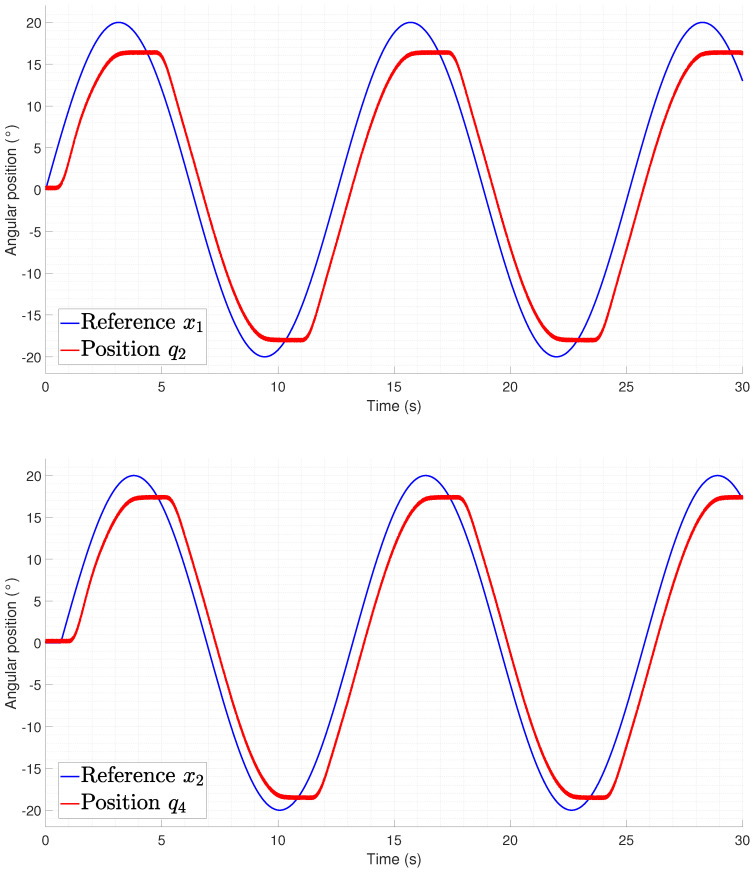
Tracking of pair joints during a lateral undulation gait pattern ($q_{2},q_{4}$).

**Figure 30:**
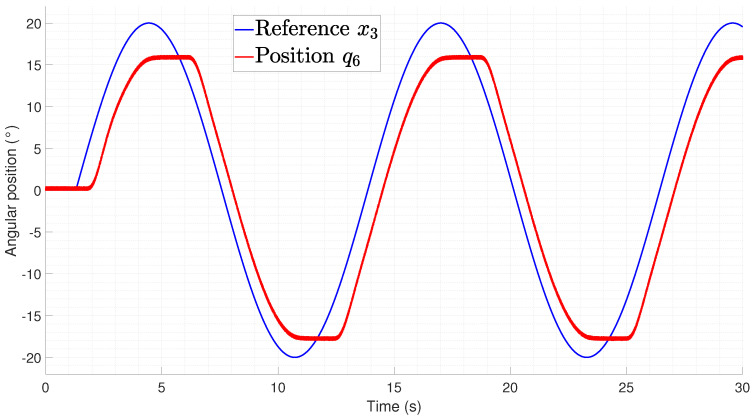
Tracking of pair joints during a lateral undulation gait pattern ($q_{6}$).

The rectilinear crawling tracking performance is shown in Figure 29 and Figure 30 from which it can be seen that the difference between the experiments performed on the lateral undulation was the delay time required to initialize the motion from the joints. This is related to the gravitational component that must be compensated for by the actuators, thus meaning that an increase in the temperature is required for the spring to produce a force.

To evaluate the performance in the tracking task, a performance integral square error (*ISE*) index was proposed, which is defined as follows:

(11) $$ISE=\int_{0}^{t}e^{2}dt.$$

The *ISE* values for the tracking tests are given in Table 3, which allows us to notice that the robot presented better tracking performance when lateral undulation was performed. The increase in the scale of the *ISE* for the rectilinear crawling was related to the gravity applied over the joints, thus increasing the delay time in tracking. This was noticed due to the fact that the delay when the error was negative was lower than in the cases where the joint was required to perform a motion to compensate for the effect of gravity.

## 7. Conclusions

The developed robotic system provided important information regarding the feasibility of implementing snake robots that use SMA actuators to generate a compact structure that could feasibly be implemented for tasks performed in reduced spaces. The conclusions of this work can be summarized as follows:

- The use of SMA spring-shaped actuators is a feasible way to actuate a snake robot that requires a reduced structure without the necessity of implementing a mechanical transmitter system.
- The measurement of the temperature on the SMA actuators is an unavoidable necessity to ensure that they are maintained in the functioning range to avoid damage to the mechanical structure or modifications of their shape programming.
- The estimation of angular positions of the Cardan joint can be performed using an array of two Hall effect sensors in this kind of structure, thus ensuring that the motion of the joints is correctly performed.
- The problem related to the multiple measurements required for the sensing of the temperature and position on the robot can be solved through the implementation of analog multiplexers, thus reducing the number of data acquisition channels required.
- The signal references required to perform motions such as rectilinear crawling or lateral undulation could be generated through the implementation of a CPG based on synchronized classical oscillators.
- The emulation of the change in snake shape could be performed using a mechanical structure based on Cardan joints; however, this requires increasing the number of links to improve the capacity to generate shapes with sufficient curvature.
- The way in which the Cardan joints are implemented allows for easy access to the SMA actuators, thereby enabling substitution once their degradation reduces the mobility of the robot.
- There exists a drawback related to the response time from the actuators with respect to the tracking of a continuous reference. As such, further research should be conducted to design an approach for compensation of the response speed.

## Figures and Tables

**Table 1:** Physical properties of the nitinol.

Property	Value	Units
Resistance	8.3	[Ω/m]
Density	6.45	[g/cm^3^]
Specific heat coefficient	0.2	[cal/°·g]
Convection heat coefficient	0.18	[W/°·g]

**Table 2:** Denavit–Hartenberg parameters for the proposed snake robot.

*i*	$a_{i}$	$\alpha_{i}$	$d_{i}$	$\theta_{i}$
1	0	$\frac{\pi }{2}$	0	$\theta_{1}^{*}$
2	*l*	$-\frac{\pi }{2}$	0	$\theta_{2}^{*}$
3	0	$\frac{\pi }{2}$	0	$\theta_{3}^{*}$
4	*l*	$-\frac{\pi }{2}$	0	$\theta_{4}^{*}$
5	0	$\frac{\pi }{2}$	0	$\theta_{5}^{*}$
6	*l*	$-\frac{\pi }{2}$	0	$\theta_{6}^{*}$

**Table 3:** *ISE* index values.

Motion	Joint	ISE
Lateral undulation	1	6585.8
Lateral undulation	3	4063.8
Lateral undulation	5	8530.5
Rectilinear crawling	2	11,591.2
Rectilinear crawling	4	8616.2
Rectilinear crawling	6	12,171.7

## Data Availability

Data are contained within the article.
